# PBPK Modeling to Support Bioavailability and Bioequivalence Assessment in Pediatric Populations

**DOI:** 10.1007/s11095-025-03846-y

**Published:** 2025-03-26

**Authors:** Fang Wu, Eleftheria Tsakalozou, Gilbert J. Burckart, Rebeka Žakelj, Lu Gaohua, Kazuko Sagawa, Viera Lukacova, Siva Vaithiyalingam, Jianghong Fan, Nikoletta Fotaki, Nikunjkumar Patel, Lanyan Fang

**Affiliations:** 1 Office of Research and Standards (ORS), Office of Generic Drugs (OGD), Center for Drug Evaluation and Research (CDER), U.S. Food and Drug Administration (FDA), Silver Spring, MD USA; 2 Office of Clinical Pharmacology (OCP), Office of Translational Sciences (OTS), CDER, U.S. FDA, Silver Spring, MD USA; 3 Lek Pharmaceuticals d.d., a Sandoz Company, Ljubljana, Slovenia; 4 Bristol Myers Squibb, Princeton, NJ USA; 5 Pfizer Global Research and Development, Groton, CT USA; 6 Simulations Plus, Inc., Lancaster, CA USA; 7 Regulatory Affairs, Cipla LTD, Warren, NJ USA; 8 University of Bath, Bath, UK; 9 Certara UK Ltd, Sheffield, S1 2BJ UK

**Keywords:** Absorption, PBPK modeling, Pediatrics, Virtual bioequivalence

## Abstract

This report summarizes the proceedings for Session 3 of the one-day public workshop entitled “Advances in PBPK Modeling and its Regulatory Utility for Oral Drug Product Development” a jointly sponsored workshop by U.S. Food and Drug Administration (FDA) and the Center for Research on Complex Generics (CRCG) on October 12, 2023. The theme of this session was the application and relevant considerations for PBPK modeling in supporting bioavailability (BA) and BE assessment in pediatric populations. The takeaway message from this session was that PBPK modeling can support relative BA and BE assessment in pediatrics since such studies are generally performed in adults or healthy subjects. PBPK absorption modeling can incorporate characteristics of the drug substance and formulation as well as pediatric physiology to assess the potential differences in absorption of different formulations in pediatrics for new and generic drugs. It is necessary to consider the totality of data and use all available evidence integrated into a mechanistic PBPK model to support decision-making. Global research efforts are needed to bridge critical data gaps.

## Introduction

The U.S. Food and Drug Administration (FDA) and the Center for Research on Complex Generics (CRCG) held the workshop titled “Advances in PBPK Modeling and its Regulatory Utility for Oral Drug Product Development” on October 12, 2023 [1]. Day 1 Session 3 of this workshop focused on the application of PBPK absorption modeling in pediatric regulatory submissions, the latest advancements and challenges for assessing relative bioavailability (BA) and BE, and to support the development of pediatric drug products. This session also discussed the development of biopredictive *in vitro* dissolution for pediatric products and to support assessments of BA and BE in pediatric populations. A thorough panel discussion followed these presentations in which the panel members highlighted several technical and regulatory key aspects regarding the utility of PBPK absorption model in pediatrics.

Relative BA is a term used in the context of Investigated New Drugs (INDs) and New Drug Applications (NDAs) to compare the BA of different drug formulations, such as pediatric and adult formulations. Demonstrating BE may not be necessary as new drugs have the potential for more significant changes in the formulations in comparison to generic drugs, which are intended to be comparable to a reference listed drug. The objective of the relative BA study is to assess the relative differences in exposure between the two formulations in terms of clinical relevance. Based on dose/concentration–response data, the applicant can either justify that the differences in rate and extent of absorption do not affect the safety and efficacy of the drug product in pediatrics or propose different dosage in pediatrics. On the other hand, BE is used in the context of generic drug products [2], which are submitted in Abbreviated New Drug Applications (ANDAs). It is used to support a determination that a generic product can be substituted for its reference listed drug or reference standard in the pediatric population, with predetermined BE limits specified for comparisons between the test product and reference standard.

For the Guidance recommendations on relative BA studies which are used to bridge adult to pediatric formulation, according to International Council for Harmonization (ICH) E 11 guideline issued in December 2000, Guidance for Industry: Clinical Investigation of Medicinal Products in the Pediatric Population, relative bioavailability comparisons of pediatric formulations with the adult oral formulation typically should be done in adults [3]. ICH E11A guideline on pediatric extrapolation issued in April 2022 further provides a roadmap to aid drug developers and regulators on the degree to which pediatric extrapolation can be applied, and the information that should be collected to address gaps in knowledge supporting the safe and effective use of medicines in the pediatric population [4].

Generic drug products are expected to have the same clinical effect and safety profile when administered to patients under the conditions specified in the labeling. That includes all indications and all patient populations including the pediatric population. In a review of 12 years of BE data comparing generic and innovator drugs, the average difference in geometric mean ratios of Cmax and AUC between generic and innovator products from BE studies was within 5% [5]. This result demonstrates that overall, the differences in pharmacokinetics (PK) parameters (e.g., AUC and Cmax) is relatively small between innovator drugs and corresponding generic drugs approved for marketing in the US. In general, the FDA recommends that BE studies be conducted in healthy adult subjects and the BE conclusions in healthy adult subjects can be extrapolated to pediatric patients. If the drug product is predominantly intended for use in pediatric patients younger than 6 years, the applicant should justify that the BE study results obtained from adult subjects are relevant to the pediatric population [6]. FDA recommends that this justification include information supporting that the inactive ingredients in the proposed products are appropriate for use in the pediatric population [6]. PBPK modeling could provide prediction results for the use of the proposed drug product in pediatrics and can be used as one of the justifications and/or to add more confidence in extrapolating adult BE results to pediatric population. This workshop session intensively discussed the application of PBPK absorption modeling in pediatric regulatory submissions and extrapolation of relative BA and BE results from adults to pediatrics.

**Summary of Presentation “Regulatory Applications and Research of Absorption Modeling for Pediatric Products”** by Dr. Lanyan Fang, Deputy Director of Division of Quantitative Methods and Modeling from the Office of Research and Standards (ORS), Office of Generic Drugs (OGD), Center for Drug Evaluation and Research (CDER), U.S. FDA [1]

Dr. Fang’s talk centered around three key points:1) How to ensure that potential differences in absorption of different formulations in pediatric patients are appropriately translated from adult data? 2) How do we identify drug products where we should be cautious? 3) What would be our approach if high risk products are identified?

From a new drugs perspective, PBPK modeling and simulation has been broadly applied to address pediatric clinical pharmacology issues. The number of pediatric PBPK models in NDA submissions has remained constant at about 5%−15% over the year of 2008 to 2023 [7, 8]. The main intended application of a pediatric PBPK model is to predict PK and propose an initial dosing recommendation for pediatric trials. For children younger than 2 years old, the PBPK approach for predicting PK may be preferred over an allometric scaling approach, especially in cases where ontogeny is an important determinant of a drug’s absorption, distribution, metabolism, and excretion (ADME). There are also cases where PBPK modeling was used to predict relative BA in pediatrics prior to pediatric trial initiation [9]. Additionally, pediatric PBPK models have been used to predict drug drug interactions (DDIs) in the pediatric population, where conducting a clinical PK study is not feasible [10].

Recently, PBPK has been used to assess pediatrics food effect (FE) [11]. FE studies are conducted in healthy adults during drug development to characterize food-drug interactions which may alter efficacy or safety of the drug. However, pediatric patients have substantial physiological, and dietary differences from adults which may cause FE that may not be translatable from adult FE information. Dr. Gilbert Burckart and his group conducted research to identify oral drug products for pediatric use with FE observed in adults using publicly available data and to summarize the implications in pediatric patients < 6 years of age [12].

Dr. Fang’s presentation focused on FDA’s proactive research efforts to ensure that BE established in adults are relevant for pediatric generic drugs [13]. The FDA contracted with University of Birmingham to evaluate risk mitigation in the evaluation of relative BA of pediatric generic products. The developed risk mitigation tools were based on the Biopharmaceutics Classification System (BCS), biorelevant *in vitro* dissolution testing and PBPK modeling [14].

To identify potential situations where a BE study performed in healthy adult subjects does not directly translate to pediatrics, a better understanding of the developmental changes across the pediatric age groups is needed. For instance, the age-related absorption changes in terms of gastrointestinal (GI) physiological differences, such as GI motility, GI fluid volume and composition and transit time need to be taken into account. In addition to age-related changes in physiological parameters affecting drug absorption, some characteristics of Active Pharmaceutical Ingredients (API)s and formulations may also play a role [13]. PBPK models can serve as a tool to evaluate the interplay between the population related factors and API/formulation related factors. A well-established PBPK modeling approach should help identify mechanisms potentially causing differences in absorption in pediatrics as compared to adults. In fact, there are published examples of validated PBPK models with good predictive performance in terms of BE outcome in adults with pediatric formulation [14, 15]. Dr. Fang concluded that absorption modeling can incorporate characteristics of the drug substance and formulation as well as pediatric physiology to evaluate the impact of formulation differences on absorption between adults and pediatric patients for new and generic drugs.

**Summary of Presentation “ Is Pediatric Absorption Modeling Ready for BE Assessment?”** by Dr. Lu Gaohua, Senior Director, Head of PBPK, Bristol Myers Squibb [1]

Dr. Lu noted the increasing utilization of PBPK in advancing biopharmaceutics necessitates mechanistic modeling of oral absorption. In this presentation, the speaker identified three fundamental questions: 1) Have we made the right assumptions in oral absorption models? 2) Do we have the right data for pediatric absorption modeling? and 3) Are we using the right oral PBPK model for locally acting drugs? By emphasizing potential discrepancies and providing conservative (not necessarily negative) responses to these queries, the speaker underscored the cautionary message that the expectation of performing “BE assessment” with pediatric PBPK mechanistic oral absorption (MOA) models may need more efforts in generating suitable data to parameterize the models, improving the models to better characterize adult *vs* pediatric differences in oral absorption and performing more validation of the models to build confidence.

**Fig. 1:**
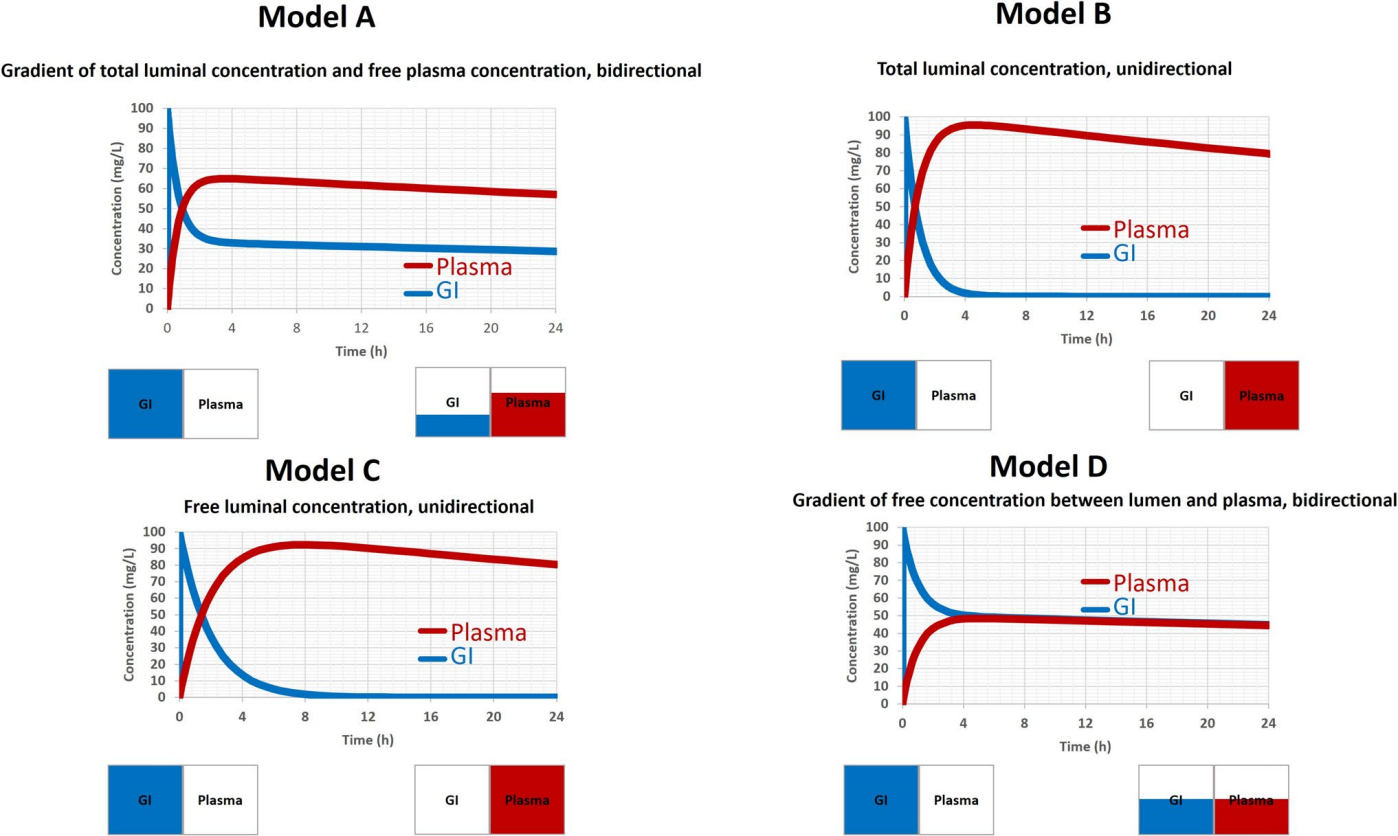
Absorption simulations based on four mechanistic oral absorption (MOA) models.

The presentation explored the assumptions in the passive permeation of drugs across the GI membrane within the framework of four MOA models: GastroPlus Advanced Compartmental Absorption and Transit (ACAT) model, Simcyp Advanced Dissolution, Absorption, and Metabolism (ADAM) model with standard Peff, Simcyp ADAM model with Mech-Peff, and Simcyp Multi-layer gut wall within ADAM (M-ADAM) model. The speaker pointed out that the Simcyp M-ADAM model has integrated the concentration gradient between free luminal concentration and free enterocyte concentration based on the pH-partition hypothesis to drive passive intestinal absorption/exsorption, while the other two Simcyp ADAM models rely on total luminal concentration and uni-directional flux and the GatroPlus ACAT model defines the passive absorption based on difference between total luminal concentration and free enterocyte concentration (Table I). Lacking proper implementation of pH-partition hypothesis in these three MOA models carries substantial consequences for absorption simulations (Fig. 1).

**Table I:** Assumptions in the Passive Permeation of Drugs Across the Gastrointestinal (GI) Membrane within Four Mechanistic Oral Absorption (MOA) Models

Oral Absorption Model	Passive absorption	Assumptions	Model
GastroPlus ACAT model	Difference between Total luminal concentration and Free enterocyte concentration	Bi-directional (Non-sink condition)	A
Simcyp ADAM model with standard Peff	Total luminal concentration	Uni-directional (Sink condition)	B
Simcyp ADAM model with Mech-Peff	Free luminal concentration	Uni-directional (Sink condition)	C
Simcyp M-ADAM model	Difference between Free luminal concentration and Free enterocyte concentration	Bi-directional (Non-sink condition)	D

As shown in Fig. 1, total concentrations in gastrointestinal (GI) and plasma were simulated using a conceptual model with GI and plasma compartments that interact through passive diffusion only. The two compartmental model conceptually represented the four oral absorption models in Table I. For a 100 mg oral dose administered into the GI compartment, using Model D (corresponding to the Simcyp M-ADAM model), which defines a bi-directional passive diffusion based on the gradient of free concentration between GI and plasma, the drug was absorbed from GI into the plasma, leading to a decrease in GI concentration and an increase in plasma concentration over time. Eventually, equilibrium between both compartments was reached. In contrast, with Model B or C (corresponding to the other two Simcyp ADAM models), although the drug was assumed to be 100% in the GI, nearly all drugs was eventually absorbed into the plasma, despite the plasma drug concentration being much higher than that in the GI. This was due to the reason that both Model B and C assume a uni-directional absorption (a sink condition) from GI to plasma. While with Model A, there was no equilibrium between the GI and plasma compartments, and plasma drug concentration can remain higher than that in GI. Notably, passive diffusion in Model A (corresponding to GastroPlus ACAT model) was driven by the differences between the total luminal concentration and the free plasma concentration (bi-directional). Compared to Model D, Model A, B and C may overestimate passive absorption. The presentation then centered on addressing the data deficiencies crucial for parameterizing the pediatric MOA model. The speaker highlighted substantial data gaps (such as the thickness and pH of the mucus, the volume of enterocyte and lamina propria, and the ontogeny of these physiological parameters) pertaining to pediatric GI physiology, not only in the Caucasian population but also in Chinese and Japanese populations. The acknowledgment of data gaps emphasizes the challenges in extending the PBPK model from adult to diverse pediatric populations and underscores the necessity for global research efforts to bridge these critical data gaps.

The presentation further illustrated the advantage of Simcyp M-ADAM model through a real case involving intravenous theophylline detoxification using activated charcoal. Intravenous theophylline, characterized by high permeability and lacking transporters affecting its intestinal absorption/exsorption, exhibited an increased systemic clearance and a shortened half-life when co-administered with oral activated charcoal [16]. Neither GastroPlus ACAT model nor Simcyp ADAM model (with standard Peff or MechPeff) could adequately analyze the observed data, highlighting the exclusive capability of Simcyp M-ADAM model, which incorporates the appropriate mechanistic details of oral absorption.

In summary, the talk emphasized the key takeaway that a robust PBPK MOA model necessitates right assumptions, right data, right model structure, and right expectations (absorption, distribution, metabolism and excretion, ADME for PBPK).

**Summary of Presentation “PBPK Absorption Modeling and Virtual Bioequivalence Assessment to Support a Pediatric Formulation Regulatory Submission”** by Dr. Kazuko Sagawa, Research Fellow, Pfizer Global Research and Development [1, 17]

Tofacitinib is a potent and selective inhibitor of the Janus kinase (JAK) family of kinases with a high degree of selectivity within the human genome’s set of protein kinases 1 [18]. Inhibition of the JAK dependent cytokines are known to have a role in multiple inflammatory diseases such as rheumatoid arthritis (RA), psoriatic arthritis (PsA), ulcerative colitis (UC), ankylosing spondylitis, and psoriasis. Currently approved formulations for Xeljanz (tofacitinib citrate salt) are immediate release (IR) tablets, modified release (MR) tablets and IR solution. IR solution was developed to enable weight-based dosing in pediatrics and was approved for polyarticular course juvenile idiopathic arthritis (pcJIA). A pediatric MR microsphere formulation was developed to enable once daily (QD) dosing in pediatric patients for the treatment of pcJIA.

A Simcyp PBPK model was developed and verified with various formulations and multiple dosing regimen for tofacitinib. The tofacitinib absorption model was built with parameters based on physiological principles and *in vitro* measured values. Model verification was performed by comparing the predicted *vs* observed clinical data from various single dose and multiple dose PK studies and food effect studies. The results of the model verification demonstrated the validity of model input parameters in predicting PK profiles of various formulations under fasted and fed conditions [17].

To bridge the efficacy and safety of the IR solution to the MR microsphere formulation, demonstration of BE between the twice a day (BID) IR solution and once daily (QD) MR microsphere formulation was needed. The extensive prior PK understanding as has been captured in the verified PBPK model provided an opportunity to conduct virtual BE (VBE) trials in lieu of a traditional clinical BE study in healthy volunteers. This model-informed approach was proposed to and accepted by both FDA and EMA [17]. It was noted in this work that VBE trials within Simcyp tool with within subject variability (WSV) are unable to replicate the clinically observed intra-subject coefficient of variation (ICV). Therefore, a novel simulation approach was developed and applied to conduct VBE by integrating PBPK model generated PK data with prior knowledge of clinical observed ICV [19].

In the study referenced above, a sample size for VBE trials was calculated based on the predetermined criteria of possibility of success (POS) and the random variability estimated as the ICV from completed tofacitinib clinical trials. The assumption of geometric mean ratio (GMR) between IR *vs* MR of 1.10 was used. The GMR of 1.10 was a conservative choice to account for the possibility of differences between IR and MR formulations. The sample size choice of 24 which corresponds to a higher POS was selected to account for any additional or unanticipated variability in the MR formulations.

For VBE assessment, the PK profiles were generated in 5000 subjects each administered the IR solution and MR microsphere formulation using the verified PBPK model. Twenty-four individuals were randomly selected from the 5000 individual pool and 1000 VBE trials were conducted to estimate the POS for BE. The VBE conducted with the verified PBPK model after incorporating clinical variability for tofacitinib demonstrated an almost negligible risk of BE failure between the IR solution and MR microsphere formulation. In conclusion, this presentation demonstrates an innovative approach that incorporates clinically observed ICV in PBPK model based VBE.

## Panel Discussion

To discuss the current thinking and knowledge gaps regarding the use of mechanistic modeling approaches to support BE assessments for pediatric products, a panel discussion was conducted which included representatives from regulatory agencies, industry, and academia. The panel discussion was moderated by Gilbert Burckart, Pharm D (Associate Director for Pediatrics, OCP, OTS, CDER, FDA) and Rebeka Jereb, PhD (Scientist, Lek Pharmaceuticals d.d., a Sandoz Company, Ljubljana, Slovenia). Sivacharan Kollipara, M Pharm (Team Lead, Biopharmaceutics, Dr. Reddy’s Laboratories); Jianghong Fan, PhD (Division of Pharmacometrics, Office of Clinical Pharmacology, Office of Translational Sciences, CDER, FDA); Lanyan Fang, PhD (Deputy Director of DQMM, ORS, OGD, CDER, FDA); Nikoletta Fotaki, MSc, PhD, FAAPS (Professor of Biopharmaceutics, CTI, University of Bath); Lu Gaohua, PhD (Senior Director, Head of PBPK, Bristol Myers Squibb); Viera Lukacova, PhD (Chief Scientist, Simulations Plus, Inc.); Nikunjkumar Patel, PhD (Senior Director of PBPK Consultancy, Certera Inc); and Kazuko Sagawa, PhD (Research Fellow, Pfizer Global Research and Development) participated in the discussion panel. In the following part of this report, we have summarized the questions raised and discussed based on the entire panel discussion.

- **Question 1**
  *Can relative BA study with pediatric delivery vehicles (such as soft food, baby formula) conducted in adults (using pediatric formulations) serve as a basis for pediatric product label? Can PBPK modeling be leveraged for this concern?*
  It may be premature to develop labeling language based solely on modeling and simulation predictions. However, through the PBPK modeling approach, we can identify knowledge gaps and outline a path for increasing model credibility in pediatric populations. For pediatric products, there are potential safety risks with respect to the impact of excipient and food on *in vivo* PK. For low-risk products, a relative BA study conducted in adults with pediatric delivery vehicles may be able to serve as a basis for pediatric product label. When a new age-appropriate pediatric formulation is developed, the applicant should conduct a new food effect study with the pediatric formulation in adults. These results can then be applied to the pediatric population. Applicants can use foods and quantities of food that are commonly consumed with drugs in a particular pediatric population (e.g., formula or milk for infants as well as jelly, pudding, or apple sauce for toddlers) [20].
- **Question 2**
  *How confident are we to conduct PBPK absorption modeling in the age of < 3 months to 4 years old without clinical data (by extrapolation from the age group of 4 years and above)?*
  To extrapolate the observed BE outcome between two different formulations from adults to 3-month to 4-year-old children, it is critical to understand the absorption mechanism of the formulations to build a model based on the fundamental properties of formulations, and importantly, to verify the model with available clinical data. Modeling and VBE simulations can help in making a risk assessment/decision about dosing in pediatrics or quality control for pediatric products for the age group of 6 to 18 years of age. It is more challenging to predict PK with PBPK modeling for the age group below 6 years old due to lack of clinical data to verify the model, as well as lack of information on physiological conditions including ontogeny profiles. For the age group below 2 years, when clinical data is available, population PK modeling is used for further interpretation and extrapolation of the PK data, and literature could also help understand different physiology in pediatrics. The panel acknowledges that there are gaps for the extrapolation in this young cohort with PBPK modeling approach. The available data on physiological and biological parameter values in this young cohort are still sparse. Artificial intelligence may help gather the data and analyze.
- **Question 3**
  *What were the experimental conditions for in vitro testing such as the buffer volume and pH? Are they considered or adjusted for Pediatrics?*
  Currently, experiences are evolving in terms of biorelevant media based on the physiological characteristics in children. According to a research using carbamazepine tablets as an example [14], the composition of the dissolution medium may not have significant impact on dissolution. On the other hand, dissolution medium volume, e.g., adjusted from 500 ml for adults to 200 ml for pediatrics plays a critical role in mimicking pediatric situation. This research incorporated the biorelevant dissolution data into PBPK model and used the model and virtual BE simulations to support relative BA and BE assessment in pediatrics.
- **Question 4**
  *Can we leverage BCS classification for risk assessment of pediatrics products?*
  Yes, we can. By recognizing the age dependent physiological differences between the adults and pediatrics and developing a pediatric BCS, we can certainly use this for risk assessment for pediatric products. Between 2011 and 2014, an interagency working group between NIH and FDA, evaluated all pediatric products that were available at that time and analyzed BCS classifications [21]. In addition, in a workshop supported by FDA and Product Quality Research Institute (PQRI) in February 2024, pediatric formulations and PBPK applications were also discussed [22]. The panel recognized that for defining pediatric specific BCS, permeability is more challenging. Therefore, it would be desirable to allocate resources on characterizing permeability in pediatric populations. In short, the panel agreed to leverage BCS classification for risk assessment of pediatric products.

## Summary of In‐Person Round Table Discussion

Following the hybrid (virtual and in-person) sessions, a dedicated in-person round table discussion session included representatives from regulatory agencies, industry, and academia. This session focused on the application of PBPK absorption modeling in pediatric regulatory submissions, the latest advancements and challenges for assessing relative BA and BE, and to support the development of pediatric drug products. This session was moderated by Lanyan Fang, PhD (Deputy Director, DQMM, ORS, OGD, FDA) and Viera Lukacova, PhD. (Chief Scientist, Simulations Plus, Inc.). This discussion addressed critical questions regarding the utilization of PBPK models for predicting drug exposure and PK in pediatrics and discussed the challenges in this area as well as the consideration points.

- *Q1: What are the considerations for making the right assumptions in a PBPK absorption model for pediatrics in terms of system parameters, e.g., pediatric GI physiology parameters as well as input parameters (e.g., compound and formulation specific parameters)?*
  The group discussed the following consideration points: 1) When using a PBPK model to predict BA or BE, the impact of disease on GI physiology may need to be considered. In addition, enzyme (metabolism/transporters) ontogeny in the pediatric population and the disease impact on activity may need to be characterized because in many cases these parameters are influential to predicted outcomes; 2) High variabilities in pediatric PK data are observed even in very well controlled *in vivo* studies. Considering the higher inter-individual variability in pediatrics compared to adults and the fact that currently the *in vivo* BE studies for pediatric formulation are mostly in adult subjects, the session participants discussed whether the BE criteria of 90% of T/R ratio falling within 0.80–1.25 in adults should be re-evaluated and less stringent criteria may be used in BE simulations with pediatric (and virtual) subjects. The session participants agreed that further research and discussions are necessary before a conclusion can be reached on this topic; 3) GI physiology of neonates is quite different than the other age groups, therefore, caution is needed when extrapolating the PK results from adults to neonates; 4) The impact of food on the absorption of drug in pediatrics may need to be considered. Note that the type of food could vary in pediatric populations participating in *in vivo* studies for BA or BE, e.g., yogurt *vs* apple sauce or other juices. The composition of these foods also differs from one country/region to another, adding to the observed population variabilities. The stability of active pharmaceutical ingredients in food or infant/baby formula is an important factor to consider. Considering the binding of drug to food contents, mechanistic PBPK modeling may be utilized to incorporate the binding of drug with excipients or food. In addition, fed and fasting states may be conducted under distinct conditions in the different pediatric populations. For instance, in neonates/toddlers, fasting state may not be feasible and realistic. Similarly, gut physiology (transit time, pH, etc.) may differ, and relevant physiological processes such as stomach emptying or gallbladder emptying may be triggered by food in an inconsistent manner in these age groups; 5) Dose is administered in formula in neonates and infants. The administration can take over 20 min. As a consequence, the PK profile cannot be extrapolated from bolus dose in adults or young pediatric cohorts; 6) There is a knowledge gap regarding the interplay between physiology in pediatrics and formulations, which needs further research.
- *Q2: In what situation is predicted pediatric pharmacokinetics (PK) acceptable by using the model developed/validated based on adult clinical data but without pediatric clinical data?*
  The FDA has accepted PBPK modeling with other supporting evidence for BE assessment in pediatric populations in some limited cases [9]. The assessment steps have been developed under scientific and regulatory considerations that include but are not limited to 1) Explore the fraction absorbed of the drug product and identify the risk factors that may result in non-BE. Challenges may exist to identify factors that impact absorption and differ between adults and pediatrics; 2) Use mass balance data to understand ADME process of the drug; 3) Use drug-drug interaction (DDI) studies with proton pump inhibitor (PPI) and food effect studies in adults to understand the mechanism of food effect, then consider food composition, feeding habits and drug vehicle and how these factors will impact drug absorption in pediatrics; 4) Dissolution testing may provide useful information to help understand the related risks and PBPK modeling can help conduct the risk assessment. The discussion group members also mentioned that BCS can be linked with the risk assessment. According to the panelists, the currently acceptable PBPK cases for pediatric products by the FDA include BCS class I drugs of reduced risk or weakly acidic BSC Class II drugs that demonstrate higher solubility at intestinal pH [9]; 5) Consider age-dependent changes in the physiology and conduct sensitivity analysis to evaluate the impact of these physiology factors on ADME in pediatrics *versus* in adults; 6) Understand PKPD relationship in adults and pediatrics. Pediatrics dose response relationship may be different than the one in adults. When utilizing model predictions to extrapolate BE results from adults to pediatrics, differences in dose–response relationships may need to be considered; 7) When determining the dose in clinical studies in pediatric populations, slowly titrating the dose up while considering the adverse events was suggested. The session participants agreed that it is necessary to consider the totality of data and use all available evidence integrated into a mechanistic PBPK model to support decision-making.
- *Q3: Could you please share your experiences on using PBPK model for GI locally acting drugs for pediatrics?*
  The amount of drug (both dissolved and undissolved) in local gastrointestinal (GI) tract would need to be considered for BE assessment. The biggest challenge in this area is that the local drug bioavailability is unknown, which makes it difficult to develop and validate PBPK models. There is limited knowledge on GI physiology in pediatrics as well. Another challenge is that limited research is available for understanding the pharmacologically optimal drug concentration in mucus or lamina propria. While PBPK models do have abilities to predict drug concentration/amount in gut lumen and enterocytes, currently, many of the MOA models in commercially available PBPK platforms do not have the capability to accurately predict drug amounts in the mucus or lamina propria. As such, it would be challenging to use these MOA models to develop appropriate pediatric PBPK models to support BE assessment for the locally acting drugs that target mucus or laminal propria.
- *Q4: What are the future advancements of PBPK modeling tools to incorporate complex pediatric dosage forms including taste masking technologies?*
  Pediatric drug product formulations may need to be modified to be taken by children, e.g., adding taste masking excipients or colorants, and sometimes crushing is also needed to facilitate drug administration in children. These modifications and the impact of excipients/vehicle on drug absorption may need to be considered when using modeling approaches. For example, enteric coating may alter absorption considering the release may be impacted by the co-administered food. In addition, excipients may have a different effect on GI physiology (motility, etc.) in pediatrics compared to in adults [23]. Children may be more or less sensitive to the excipients’ effects than adults. PBPK modeling can be utilized to assess the impact of excipients on gut absorption by incorporating passive permeability and/or the impact on transporter mediated transport as well as on drug metabolism in the gut. Recent GDUFA funded research has aimed to understand the excipient impact on permeation for BCS class III drugs using *in vitro* systems and *in vivo* data in humans [24, 25]. These *in vitro* testing results may be incorporated into mechanistic PBPK modeling to predict the risk for non-BE and link *in vitro* testing results to *in vivo* performance.

## Conclusion

This workshop session discussed recent advances in using PBPK absorption modeling for relative BA and BE assessment for pediatric drug products. During the workshop, successful cases and views on the best practices, challenges, and opportunities for the utility of PBPK absorption modeling in these areas were shared. The takeaway message from this session was that PBPK modeling can support relative BA and BE assessment in pediatrics since such studies are generally performed in adults or healthy subjects. Mechanistic PBPK absorption modeling can incorporate characteristics of the drug substance and formulation as well as pediatric physiology to assess the potential differences in absorption of different formulations in pediatrics for new and generic drugs. Ultimately, it is necessary to consider the totality of data and use all available evidence integrated into a mechanistic PBPK model to support decision-making for pediatric drug products. Additionally, global research efforts are still needed to bridge critical data gaps.

## Declarations

### Disclaimers

The views expressed in this article are those of the authors and should not be construed to represent the FDA’s views or policies. The mention of commercial products, their sources, or their use in connection with material reported herein is not to be construed as either an actual or implied endorsement of such products by the FDA.

## References

[1] CRCG-FDA Workshop: Advances in PBPK Modeling and its Regulatory Utility for Oral Drug Product Development. Available at: https://complexgenerics.org/education-training/advances-in-pbpk-modeling-and-its-regulatory-utility-for-oral-drug-product-development/. Accessed July 2024.

[2] National Archives and Records Administration. 21 CFR 314.94 Content and format of an ANDA. Available at https://www.ecfr.gov/current/title-21/section-314.94. Accessed Nov 2024.

[3] Guidance for Industry: E11 Clinical Investigation of Medicinal Products in the Pediatric Population. Issued in December 2000. Available at: https://www.fda.gov/media/71355/download. Accessed Sept 2024.

[4] ICH E11A Pediatric Extrapolation. Issued in April, 2022. Available at https://www.ema.europa.eu/en/documents/scientific-guideline/ich-guideline-e11a-pediatric-extrapolation-step-5_en.pdf. Accessed Sept 2024.

[5] Davit BM, Nwakama PE, Buehler GJ, Conner DP, Haidar SH, Patel DT, *et al*. Comparing generic and innovator drugs: a review of 12 years of bioequivalence data from the United States Food and Drug Administration. Ann Pharmacother. 2009;43(10):1583–97. 19776300 10.1345/aph.1M141

[6] Food and Drug Administration. Guidance for Industry: Bioequivalence Studies With Pharmacokinetic Endpoints for Drugs Submitted Under an ANDA. Issued in August 2021. Available at: https://www.fda.gov/media/87219/download. Accessed Sept 2024.

[7] Grimstein M, Yang Y, Zhang X, Grillo J, Huang SM, Zineh I, *et al*. Physiologically based pharmacokinetic modeling in regulatory science: an update from the U.S. Food and Drug Administration’s Office of Clinical Pharmacology. J Pharm Sci. 2019;108(1):21–5. 30385284 10.1016/j.xphs.2018.10.033

[8] Zhang X, Yang Y, Grimstein M, Fan J, Grillo JA, Huang SM, *et al*. Application of PBPK modeling and simulation for regulatory decision making and its impact on US prescribing information: an update on the 2018–2019 submissions to the US FDA’s Office of Clinical Pharmacology. J Clin Pharmacol. 2020;60(Suppl 1):S160–78. 33205429 10.1002/jcph.1767

[9] Amore BM, Patel N, Batheja P, Templeton IE, Jones HM, Louie MJ, *et al*. Physiologically based pharmacokinetic model development and verification for bioequivalence testing of bempedoic acid oral suspension and reference tablet formulation. Pharmaceutics. 2023;15(5):1476. 37242718 10.3390/pharmaceutics15051476 PMC10222242

[10] Salerno SN, Burckart GJ, Huang SM, Gonzalez D. Pediatric drug-drug interaction studies: barriers and opportunities. Clin Pharmacol Ther. 2019;105(5):1067–70. 30362111 10.1002/cpt.1234 PMC6465091

[11] Parrott N. Food effect in pediatric populations: current practice, challenges, and future potential for use of physiologically based biopharmaceutics modeling. J Clin Pharmacol. 2024;64(8):1044–7. 38717132 10.1002/jcph.2456

[12] Tunehag KR, George B, Samuels S, Vo K, Arya V, Abulwerdi G, *et al*. Food-drug effects and pediatric drug development studies submitted to the US Food and Drug Administration, 2012–2022. J Clin Pharmacol. 2024;64:697–703. 38294346 10.1002/jcph.2405

[13] Pawar G, Wu F, Zhao L, Fang L, Burckart GJ, Feng K, *et al*. Development of a pediatric relative bioavailability/bioequivalence database and identification of putative risk factors associated with evaluation of pediatric oral products. AAPS J. 2021;23(3):57. 33884497 10.1208/s12248-021-00592-y PMC8060189

[14] Pawar G, Wu F, Zhao L, Fang L, Burckart GJ, Feng K, *et al*. Integration of biorelevant pediatric dissolution methodology into PBPK modeling to predict in vivo performance and bioequivalence of generic drugs in pediatric populations: a carbamazepine case study. AAPS J. 2023;25(4):67. 37386339 10.1208/s12248-023-00826-1

[15] Miao L, Mousa YM, Zhao L, Raines K, Seo P, Wu F. Using a physiologically based pharmacokinetic absorption model to establish dissolution bioequivalence safe space for oseltamivir in adult and pediatric populations. AAPS J. 2020;22(5):107. 32779046 10.1208/s12248-020-00493-6

[16] Berlinger WG, Spector R, Goldberg MJ, Johnson GF, Quee CK, Berg MJ. Enhancement of theophylline clearance by oral activated charcoal. Clin Pharmacol Ther. 1983;33(3):351–4. 6337763 10.1038/clpt.1983.44

[17] Purohit V, Sagawa K, Hsu HJ, Kushner J, Dowty ME, Tse S, *et al*. Integrating clinical variability into PBPK models for virtual bioequivalence of single and multiple doses of tofacitinib modified-release dosage form. Clin Pharmacol Ther. 2024;116:996–1004. 38797995 10.1002/cpt.3313

[18] Dhillon S. Tofacitinib: a review in rheumatoid arthritis. Drugs. 2017;77(18):1987–2001. 29139090 10.1007/s40265-017-0835-9

[19] Saadeddin A, Purohit V, Huh Y, Wong M, Maulny A, Dowty ME, *et al*. Virtual bioequivalence assessment of ritlecitinib capsules with incorporation of observed clinical variability using a physiologically based pharmacokinetic model. AAPS J. 2024;26(1):17. 38267790 10.1208/s12248-024-00888-9

[20] Food and Drug Administration. Guidance for Industry: Assessing the Effects of Food on Drugs in INDs and NDAs — Clinical Pharmacology Considerations. Issued in June 2022. Available at: https://www.fda.gov/media/121313/download. Accessed Sept 2024.

[21] Intra-Agency Agreement Between the Eunice Kennedy Shriver National Institute of Child Health and Human Development (NICHD) and the U.S. Food and Drug Administration (FDA) Oral Formulations Platform—Report 1. Available at: https://www.nichd.nih.gov/sites/default/files/inline-files/Formulations_Platform_Report1.pdf. Accessed July 2024.

[22] PQRI Workshop: MIDD Approaches in Pediatric Formulation Development. Available at: https://pqri.org/pqri-pediatric-workshop-2024/. Accessed July 2024 10.1208/s12248-026-01217-y 41803558

[23] Adkison K, Wolstenholme A, Lou Y, Zhang Z, Eld A, Perger T, *et al*. Effect of sorbitol on the pharmacokinetic profile of lamivudine oral solution in adults: an open-label, randomized study. Clin Pharmacol Ther. 2018;103(3):402–8. 29150845 10.1002/cpt.943 PMC5836851

[24] Zou L, Ni Z, Tsakalozou E, Giacomini KM. Impact of pharmaceutical excipients on oral drug absorption: a focus on intestinal drug transporters. Clin Pharmacol Ther. 2019;105(2):323–5. 30663035 10.1002/cpt.1292

[25] Zou L, Pottel J, Khuri N, Ngo HX, Ni Z, Tsakalozou E, *et al*. Interactions of oral molecular excipients with breast cancer resistance protein. BCRP Mol Pharm. 2020;17(3):748–56. 31990564 10.1021/acs.molpharmaceut.9b00658 PMC8177814

